# A Case of Transient Ventilation Difficulty Caused by the Occlusion of a Nasotracheal Intubation Tube Due to the Weight of the Tongue in a Patient With Morbid Obesity

**DOI:** 10.7759/cureus.68488

**Published:** 2024-09-02

**Authors:** Naoko Tachi, Aiji Sato-Boku, Yoko Okumura, Mayumi Hashimoto, Masahiro Okuda

**Affiliations:** 1 Department of Anesthesiology, Aichi Gakuin University, Nagoya, JPN

**Keywords:** tongue weight, airway obstruction, nasal intubation, airway management, obesity

## Abstract

In patients with obesity, airway obstruction is more likely to occur because of the effects of gravity on the supine position and compression of the airway caused by the weight of soft tissues. This significantly increases the risk of apnea and hypoxemia. Therefore, careful airway monitoring and securing are essential after anesthesia induction and during postoperative recovery. Herein, we report a case of nasotracheal intubation tube obstruction caused by the weight of the tongue in a patient with morbid obesity during general anesthesia. This rare complication highlights the importance of careful airway monitoring and management in patients with obesity undergoing general anesthesia.

## Introduction

Obesity presents significant challenges in airway management during general anesthesia, primarily due to the increased soft tissue mass and altered airway anatomy associated with this condition. These anatomical changes can lead to a higher incidence of complications such as difficult intubation, airway obstruction [1], and an increased risk of perioperative morbidity and mortality [2,3]. The increased adipose tissue in the neck and pharyngeal structures, coupled with reduced functional residual capacity [4] and increased oxygen consumption, further complicates airway management in obese patients. These factors contribute to an elevated risk of hypoxemia during induction and maintenance of anesthesia.

Effective airway management in patients with obesity is critical to ensuring patient safety and minimizing the risk of adverse outcomes [5]. It requires careful preoperative assessment, meticulous planning, and the use of appropriate techniques and equipment [6] tailored to the patient's specific needs. Understanding the unique challenges posed by obesity, including the potential for airway obstruction and the difficulty of intubation, is essential for anesthesiologists.

In this report, we describe a particularly challenging case in which a nasotracheal intubation tube became obstructed due to the weight of the patient's tongue, leading to significant ventilation difficulties. This case highlights the importance of anticipating potential airway issues in obese patients and being prepared with alternative airway management strategies. The occurrence of such an obstruction underscores the need for vigilance and prompt intervention to avoid severe perioperative complications.

Written informed consent was obtained from the patient for the publication of this report.

## Case presentation

The patient was a 26-year-old woman. She was 159 cm tall, weighed 109 kg, and had a body mass index (BMI) of 43 kg/m^2^. She was scheduled for teeth extraction under general anesthesia. The patient had dental anxiety and a gag reflex. Although tooth extraction was initially planned under sedation, general anesthesia was employed because of concerns about respiratory depression during sedation. The patient had a history of obstructive sleep apnea but was otherwise healthy with no significant comorbidities. Preoperative assessment, including airway evaluation, revealed a Mallampati score of III, indicating potential difficulty in intubation. No other abnormal findings were observed in the preoperative assessment. Blood test results are shown in Table 1.

**Table 1:** Blood test WBC: white blood cell; RBC: red blood cell; Hct: hematocrit; Hb: hemoglobin; MCV: mean corpuscular volume; MCH: mean corpuscular hemoglobin; MCHC: mean corpuscular hemoglobin concentration; PLT: platelets; BUN: blood urea nitrogen; CRE: creatinine; Na: sodium; K: potassium; Cl: chloride; Ca: calcium; Tp: total protein; CK: creatine kinase; Amy: amylase; CRP: C-reactive protein; Fe: ferritin; PT: prothrombin time; APTT: activated partial thromboplastin time; INR: international normalized ratio

Complete blood cell		Biochemical test				Coagulation blood test	
								WBC	7670/uL	BS	93 mg/dl	Alb	4.1 g/dl	PT	11.5 s
RBC	498×10^4^/uL	BUN	10 mg/dl	CK	53 IU/L	APTT	28.9 s								
								Hct	41.90%	CRE	0.75 mg/dl	Amy	46 U/L	PT activity	93%
Hb	13.8 g/dL	Na	139 mmol/l	AST	18 IU/L	INR	1.04								
								MCV	84.1	K	4.0 mmol/l	A LT	21 IU/L		
MCH	28.1	Cl	106 mmol/l	γGTP	22 IU/L										
								MCHC	32.9	Ca	9.4 mmol/l	CRP	1.3		
PLT	30×10^4^/uL	Tp	7.3 g/dl	Fe	48 ug/dl										

Standard monitoring including pulse oximetry, electrocardiography, and noninvasive blood pressure monitoring was implemented. Anesthesia was induced by remimazolam because it allows for the awakening of the patient with an antagonist if mask ventilation becomes impossible after induction. Fortunately, mask ventilation was adequately achieved with a two-person technique; thus, rocuronium and remifentanil were administered.

Because of obesity and potential difficulty in airway management, a video laryngoscope was used for nasotracheal intubation. A 7.0-mm endotracheal tube (Polar™ Preformed Tracheal Tube Smith Medical Japan Ltd., Tokyo) was successfully placed through the nasal passage without initial complications. Tube placement was confirmed by capnography and bilateral breath sounds. The patient was mechanically ventilated with a volume-controlled mode. Approximately 20 minutes after anesthesia induction, the patient's oxygen saturation began to drop, from 98% to 90%, despite an increase in the inspired oxygen concentration. The anesthesiologist noted increased airway resistance and difficulty in maintaining adequate ventilation pressures. The airway was immediately evaluated.

**Figure 1:**
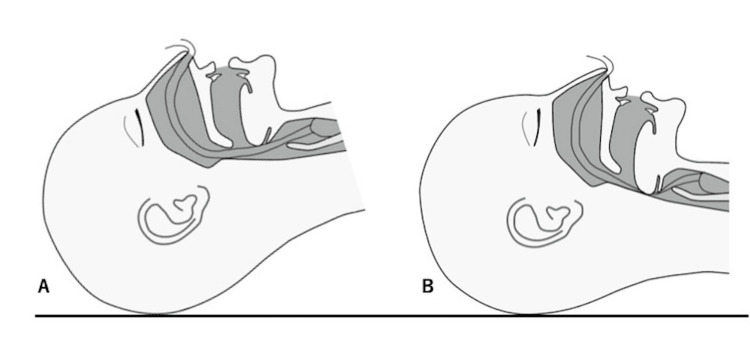
Backward head position (A) and forward head position (B) (A) The airway is open as the head is extended, and the intubation tube is unobstructed. (B) The intubation tube is obstructed by the tongue falling back associated with the flexion of the head.

Upon examination, the nasal intubation tube was compressed by the tongue, which had shifted posteriorly because of muscle relaxation and the supine position (Figure 1).

This compression partially obstructed the endotracheal tube. Immediate measures were taken to resolve the obstruction. The patient's head was repositioned into a more extended position. The nasal tube was carefully repositioned to alleviate the compression. Manual ventilation was provided to ensure adequate oxygenation and ventilation until the problem was resolved. After these interventions, the patient's oxygen saturation improved, and airway resistance decreased significantly. The surgery proceeded without further complications, and the patient was extubated uneventfully in the operating room. In the recovery room, postoperative monitoring showed stable vital signs and adequate oxygenation.

## Discussion

Studies have reported nasal endotracheal tubes becoming obstructed by blood clots, damaged middle turbinates, or foreign bodies [7,8]. However, to the best of our knowledge, no cases of obstruction caused by the weight of the tongue have been reported. This case report highlights the difficulties of airway management in patients with obesity. In particular, inappropriate selection and positioning of the intubation tube can lead to serious complications. In this patient with obesity, the excess weight of the soft tissues, particularly the tongue, compressed the nasal intubation tube inserted into the airway, resulting in partial obstruction. This led to decreased oxygen saturation and ventilation difficulties that required immediate attention. In this case, the selection of the intubation tube and anatomical changes in the airway associated with obesity must be considered.

In this patient, a 7.0-mm nasal intubation tube was used; however, given the patient's BMI (43 kg/m^2^), a tube with a larger inner diameter may be necessary. Larger tubes can decrease airway resistance and make ventilation easier. Considering that the anatomy of the nasal cavity may make insertion of a larger tube difficult and that the patient could ventilate without problems with a 7.0 tube, the tube size was not the issue, and the Polar™ Preformed Tracheal Tube was mistakenly selected. Polar™ Preformed Tracheal Tube is made of polyvinyl chloride, which is extremely soft and suitable for nasal intubation. Thus, a tube that is less prone to blockage, such as a spiral tube, should have been chosen in this case.

In patients with obesity, the tongue tends to move backward, increasing the risk of airway obstruction. In the present case, muscle relaxation caused by muscle relaxants and gravity induced by the supine position resulted in the tongue compressing the airway. This problem is specific to patients with obesity and is difficult to predict with normal intubation techniques. However, with careful attention to the head position, the patient did not experience unexpected ventilation difficulties.

## Conclusions

This case serves as a reminder of the complex challenges in the airway management of patients with obesity during anesthesia. It emphasizes the need for tailored strategies and heightened awareness to mitigate the risks of airway complications, ensuring the safety and well-being of patients with morbid obesity during surgical procedures. Anesthesiologists should be aware of the potential for nasal intubation tube obstruction because of the weight of the tongue in patients with obesity. Close monitoring and prompt interventions are crucial to ensure patient safety during anesthesia. Further studies and case reports may help develop guidelines for managing similar situations in the future.
